# Supplementary material for: Histological and metagenomic analysis of microbial communities in archaeological human bones
Source: PLoS One. 2026 May 27;21(5):e0340244. doi: 10.1371/journal.pone.0340244 (PMC13215491; doi:10.1371/journal.pone.0340244)
Supplement: S1 Appendix — (DOCX) [file pone.0340244.s001.docx]

# Site descriptions

The current paper is carried out as part of a larger, still ongoing research project (2020-2026), Future Past – *Future Preservation of past life: A multidisciplinary investigation into preservation of ancient biological remains from medieval cemeteries.* The project has carried out a research excavation in a medieval cemetery, as well as sampled museum collections in Stavanger stemming from a total of six sites. These are described in the following.

## Avaldsnes

#### General background

Avaldsnes is a significant site in Norwegian history, known as one of the longest-lasting power centres in the country, and potentially dating as far back as the Pre-Roman Iron Age (Stylegar *et al.*, 2011). This importance is due to a combination of fertile farmland and a strategic location by the narrow Karmsund Sound, a key route along the Norwegian coast that allowed ships to avoid the rough conditions of the North Sea. During the Early and High Medieval periods, the farm at Avaldsnes was owned by the king. Around 1250 AD, King Håkon Håkonsson built a church dedicated to St. Olav, which became one of 14 royal chapels in Norway (Helle, 1999), and thus part of the royal administrative system. A royal manor was most likely constructed around the same time (Nordlie and Sand-Eriksen, 2019). This manor was destroyed by the Hanseatic League in 1368. After Norway entered into a union with Denmark in 1380, the farm became the property of the church. For a long time, the St Olav chapel was the only known structure from Håkon Håkonssen’s royal farm, but in 2012 the basement of a stone-built building was uncovered, which was further investigated in 2017 (Bauer, 2017; Nordlie and Sand-Eriksen, 2019). In 2020, further excavations were carried out in connection with conservation work to improve drainage and access and visibility for the public. This revealed the presence of 30 burials in and around the tower cellar (Nordlie and Ødeby, 2021).

#### Excavation and site description

The tower, discovered in 2012 and further investigated in 2017, is interpreted as such based on the thickness of the walls. This was originally a free-standing ‘tower-like’ building, rectangular in plan, with external dimensions of ca 12,6x8,7 meters and an internal area of ca. 55 m^2^. There were no indications of a soil or stone floor, thus the building likely had a wooden floor with a crawl space. After its construction, a wall was built, connecting it to the church. The technique and style of building make it highly likely that the construction occurred around the same time as that of the extant church building, ca 1250 AD. Parts of the remaining walls had clearly been disturbed by later burials in the cemetery. Further excavations in and around the tower remains in 2020 uncovered a total of 30 burials with skeletal remains. Three skeletons were radiocarbon dated to the Medieval Period. This highlighted the difficulty in dating the burials archaeologically, as there was often no clear difference in layers and grave fills. Out of the 30 graves, 10 are clearly modern, while 7 could be assigned to the Medieval Period with some confidence. A lack of finds indicating use makes interpretation of the building difficult, but suggestions based on the known use of similar contemporary buildings are accommodation, defence, and storage of valuables. However, the discovery of graves dating to its use period suggests it cannot be interpreted as a completely secular building and that it, at least for some time, was used as a kind of chapel (Nordlie and Ødeby, 2021).

#### The material

Two individuals from Avaldsnes have been included in the study. One is a relatively complete skeleton, although missing the skull (SZ1189), whereas the other is represented only by a skull and two vertebrae (SZ1381). Both categorized as possible males (based on limited skeletal indicators), and adults of the age ranges 40-60 (SZ1189) and 17-25 (SZ1381). Radiocarbon dating results suggest that both individuals belong to the High Medieval Period (AD 1130-1350). The older individual, SZ1189, had shown evidence of healed fractures on the upper left arm and a rib. There was also evidence of active *osteomyelitis*, an infection apparent as a porosity in one of the ribs. Furthermore, the neck vertebrae displayed an increased porosity, which may be caused by tuberculosis, and thus the bacterium *Mycobacterium tuberculosis*. The skull of SZ1381 displayed osteoporotic pitting, small pits appearing on the inside of the skull. It is not known what might cause this feature. The teeth displayed some caries and heavy dental calculus. In addition, enamel hypoplasia was observed; visible lines on the teeth that indicate periods of poor enamel development, related to stress during childhood, either due to trauma or poor nutrition (Bergland, 2021).

As the building where these burials were placed was torn down in the Medieval Period, these burials have been classified as being in an ‘outdoor’ environment. Skeleton SZ21189 was found in a grave that had been partially cut into the bedrock. Skeleton SZ1381 was discovered near the door in the western wall. This section was initially opened to investigate deposits of mortar. Excavation was halted once the skull appeared, the skull and vertebrae were collected, and the trench was backfilled. It is thus possible that the rest of the skeleton is still intact *in situ* (Nordlie and Ødeby, 2021).

## Sola

#### General background

The church at Sola was built during the 12^th^ century, in Romanesque style, and is typical of rural stone churches in Norway at the time. It had a rectangular nave of 5,8 by 9,6 metres, a square choir and a square tower in the west end (Sellevold and Hommedal, 2008). The cemetery was in use until 1842, after which the church fell into ruin. In the 1870s, it was used as studio and private residence for the painter Johan Jacob Bennetter and his family. The cemetery was transformed into a garden. When the family moved out in 1907 the building was yet again left to crumble and stood without roof until the 1940s, when large parts of the walls were also torn down. Restoration work, including archaeological investigations, was carried out in the period 1982 to 1995 (Næss and Sellevold, 1990; Hommedal and Sellevold, 2017).

#### Excavation and site description

In the 19^th^ century, the soil floor and many graves inside the church had been removed to refurbish the church as an artist studio and home. The western tower floor was left untouched, however, and large parts of the cemetery were also intact.

Archaeological work focused on the floor of the western tower and an approximately 2-meter-wide trench in the cemetery surrounding the church, along the church walls. The remains of 23 individuals were found *in situ*, while the loose remains of 60 further individuals were recovered in mixed layers. Six of the graves were found in the western tower, 17 were found south of the nave, six were found east of the choir, and two were found in the nave. Thus, a minimum of 83 individuals had been buried within the excavated areas. Despite the removal of the cultural layers in the 19^th^ century, the remains of a burial chamber were also found within the nave, as well as evidence of Late Iron Age settlement, medieval church ruins, and traces of 19th-century activity. The remains of a 14th-century workshop for church bell casting were uncovered inside the church (Sellevold and Hommedal, 2008).

Although six graves were found in the west tower, the careful examination of the disturbed bones suggested that another seven individuals had likely been buried there. This collection included both sexes and all age groups from newborn to 55-60 years of age. The sequence of four of the undisturbed graves within the tower could be reconstructed based on archaeological stratigraphy. Of 31 grave cuts uncovered, only 24 were opened and 22 were fully excavated, producing an assemblage of 23 individuals as one grave (nr 4) contained two individuals. Eight of these were from burials within the church, the remaining from the surrounding cemetery. The demographic profiles of the undisturbed and redeposited remains were quite different, with a higher number of younger individuals in the disturbed than in the undisturbed assemblage, according to the excavators, suggesting that children’s graves are more often destroyed during later use of the cemetery and are thus underrepresented in the assemblages of intact *in-situ* graves (Næss and Sellevold, 1990; Sellevold and Hommedal, 2008; Hommedal and Sellevold, 2017).

The largest grave in the western tower (grave no 18) was found beneath a bell casting pit and dated to the Early Medieval period. It contained the remains of a female, around 55-60 years old. This is not a unique situation in Nordic medieval churches, as several contain a large, central grave in the western tower, often a woman. One interpretation is that these are founders’ graves, and that the women represent individuals or families that initiated and funded the building of the churches. This might also point to a central role for women in the Christianization process (Sellevold, 1996). The woman at Sola was buried in a large ironclad pine coffin. She appears to have been wrapped in some sort of textile or skin, and most likely had a pillow for her head (Hommedal and Sellevold, 1999).

#### The material

Out of the 23 excavated individuals, 17 individuals were included in the Future Past project, including five individuals from the western tower. Since the church has been an open ruin for a prolonged period, these skeletons have also been defined as ‘outdoors’, even though they were originally buried inside the standing building. The sampled remains include both males and females, and individuals from ca. 9 to 60+ years of age.

All except one of the 17 individuals have been radiocarbon dated. Five are of post-medieval date. The remaining are medieval in date, although some of these do have radiocarbon dating ranges tailing into either the Viking or post-medieval periods.

## Stavanger

#### General background

Although settlement in Stavanger does stretch back into prehistory, the record is quite patchy. It is becoming increasingly clear through relatively recent archaeological excavations that Stavanger developed as a central farm during the Viking Age, a farm where likely trading, seasonal markets and specialized crafts were carried out, where there was a pre-Christian burial ground, and where in the early medieval, before the construction of the cathedral, a Christian cemetery was established (Brendalsmo and Paasche, 2017; Høgestøl and Sandvik, 2020; Ødeby et al., 2022). With the establishment of the bishopric in the early 12^th^ century, Stavanger became the seat of one of the five dioceses in the country (Ekroll, 2013; Stige, 2013). This is also seen as the beginning of Stavanger’s history as a city. It was foremost the bishop’s city, a religious centre with an urban infrastructure of several churches, a monastery, a hospital and a school, all centred around the cathedral. A medieval cathedral did not include a cemetery for the parish, but burials would still occur, of priests, royals and nobility, and other wealthy individuals who could purchase a burial place close to the cathedral (Brendalsmo and Paasche, 2017). Burials were also carried out beneath the floor of the nave. Tombstones from the old floor show that this practice of burials inside the building started in the medieval period and it continued until it was banned in 1801 (Ødeby *et al.*, 2022).

The current cathedral building was built in two separate phases. The Romanesque cathedral, consisting of a western tower, a nave and a choir, is thought to have been built over decades, probably starting in the 11^th^ century, and completed at some point before 1150. In the mid-13^th^ century, the Romanesque choir was replaced with a much larger, Gothic choir and a monumental eastern façade (Ebert, 2024; Barrett, Ebert and Allen, 2025). The vestibule on the west front was also replaced and enlarged (Stige, 2013), where recent radiocarbon dating work has confirmed that construction took place in the 14^th^ century (Ebert, Bjelland and Philippsen, 2025).

After the Reformation, in 1537, Stavanger’s wealth and power dwindled, although the rich baroque interior testifies to the presence of a few wealthy families of priests and merchants with international ties. In 1682, the episcopal seat was moved to Kristiansand, and Stavanger was degraded to a small provincial town and port of trade, and the cathedral to a parish church (Stige, 2013). The 1600s were rough years for the town, and in addition to the loss of the episcopal seat, it experienced economic and social decline through periodic loss of trade rights, poor fishing seasons, a devastating fire and an epidemic. During the 1700s, shipping was developed into the main industry in town, and the wealth and population waxed and waned in connection with wars being fought in Northern Europe. In the 19^th^ century, the town seemed to hold a stable population with a clear social division; a large middle class and working class, and a few very rich families (Daasvand, 2012). The trade in herring, as well as the shipping, led to a boom in both wealth and population from the start of the 19^th^ century, and towards the end of it, the canning industry changed the character of Stavanger entirely. In the year 1900, the town had 30,000 inhabitants and had grown substantially beyond its old borders. The improved economic situation, the need for more space in the church, and new, national romantic ideas initiated a large restoration of Stavanger Cathedral in the period 1866 to 1874. This caused drastic changes in both the interior and exterior, but also changes in the floors and cemetery, which meant that graves (Utne, 1988) and pre-cathedral remains were removed, disturbed or destroyed (Ødeby *et al.*, 2022). The cemetery was in use till 1834, when it was turned into a park (Utne, 1988).

#### Excavations and site descriptions

##### The choir crypt

In 1967 and 1968, an 18 by 8 meter area of the crypt beneath the choir was excavated, in advance of a planned refurbishment (Rolfsen, 1968). Altogether 31 in-situ human skeletons were found, in several layers. The burials were found as clear cuts, many cutting through a layer of charcoal and burnt stones interpreted as a burnt floor. There were at least two skeletons in each cut (Rolfsen, 1968, 2021). The individuals were interred in coffins and their burials adhered to the Christian customs of east-west orientation and no grave goods. Some of the burials were cut by the foundations of the 13th-century Gothic choir.

The east-west orientation of the graves diverges from that of the cathedral itself, which lies on an east-southeast/west-southwest axis, suggesting they belong to a Christian cemetery pre-dating the construction of the cathedral. This interpretation has later been supported by radiocarbon dates. A large part of the remains became commingled during post-excavation, and the original numbering from the archaeological report was lost. The radiocarbon dates were thus carried out on right femurs, ensuring no duplicate measurements, and providing calibrated dates between ca AD 700 and 1100, with most belonging to the 11^th^ and early 12^th^ century. These results fit well with a pre-cathedral cemetery in Stavanger. While further investigations found that some of the commingled remains may stem from other sites in Norway (Denham, 2014; Høgestøl and Sandvik, 2020), most of the current commingled assemblage does likely originate from the cathedral excavation.

###### The material

In the 1960s, it was common practice to send archaeological human remains the Anatomical Institute, the University of Oslo. The remains from Rolfsen’s 1967 excavation were also sent there for analysis. The remains of nine individuals were kept in the institute’s collection; the rest returned to Stavanger Museum. At some point during this return process, the remains became commingled and dissociated from their context information. The remains were later sorted, attempts were made to reconstruct individuals based on osteometrics, and new analyses were carried out on right femurs (van der Sluis, 2012; Denham, 2014; Høgestøl and Sandvik, 2020). The collection remains problematic due to loss of information and possible commingling with material from other sites in Norway. While the remains retained at the institute in Oslo cannot be related to the information in the archaeological report, we can be confident that they stem from the 1967 excavation in Stavanger. The Future Past project has sampled skulls (the occipital bone) from nine individuals from the choir site, and so far, the radiocarbon dating of this has supported the Early Medieval dates of the choir burials.

Despite these burials being found underneath the cathedral choir, they are clearly from an outdoor cemetery pre-dating the building. For this reason, this collection has been defined as outdoor burials.

##### Stavanger Bypark (Stavanger City Park) 2005

The rescue excavation in Stavanger Byparken was carried out in 2005 in connection with the city council’s plans to refurbish the park around the cathedral (Johansen, 2005). Nine trenches were dug, trench A–I, a few meters from the cathedral walls, along the south-western side, the north-eastern side and the south-eastern side. The four skeletons included in the project (1, 2, 3 and 7, Museum numbers S12200.1-4) came from trenches A (south-eastern side) and D (southern-western side, going around the south-eastern corner), and were all located close to the south-eastern corner of the cathedral. Trench A was 18x5 meters, approximately parallel to the eastern cathedral wall, with a 5,5 meters distance to the wall, and a depth of ca 50 cm. Six layers were documented in the trench, and six in-situ skeletons in total were found in layer 2, in the south-western end of the trench. The skeletal remains were generally well preserved, but all the graves had been disturbed and were only partially intact. In-situ skeletons were also observed in deeper layers, but these were not excavated as they would not be affected by the development work. Layer 2 was described as containing a range of material, from silt to gravel and small rocks, as well as humus, noted as having a greasy and compact consistency. The soil was mixed with fragments of brick, soapstone, human bones, glass, roof slabs, porcelain, ceramics, chalk pipes and lime. These are coffin burials, although no remains of the coffins remained apart from iron rivets in the case of skeleton nr 1. Trench D was 100 m long, 90 cm wide and 60 cm deep. In situ skeletons were found in layer 4 of one profile, including skeleton number 7, just south-east of the south-eastern corner of the cathedral. Layer 4 consisted of dark greyish-brown silt and sand with mixed-in humus, some gravel and soapstone fragments with a relatively compact and medium greasy consistency. Red brick fragments were also found (Johansen, 2005)

###### The material

The four burials were all relatively shallow and younger in date. All showed varying levels of modern disturbance. Skeleton nr 1 (S12200.1), an older female (60-80 y), is missing feet, left leg and right lower arm. She displayed severe osteoporosis and was almost toothless. Skeleton nr 2 (S12200.2) consists of the left torso, left arm and left leg of an adult male (20-25 y). Skeleton nr 3 (S12200.3) was the near-complete skeleton of an adult male (30-40 y), although the skull was damaged during excavation, and the right arm and femur are missing. Skeleton nr. 7 (S12200.4), the remains of an adult female (18-22 y) were incomplete, with most of the lower body missing. The remaining bones are fragmented and in poor condition (Johansen, 2005; Sellevold, 2006).

The skeletal remains excavated from both trenches are, based on stratigraphy and location, most likely from the 1600s or 1700s (Johansen, 2005). The radiocarbon dating carried out in the Future Past project confirms this, with ranges between AD 1648-1950.

##### The nave 2021

In preparation for the city’s 900^th^ anniversary in 2025, Stavanger Cathedral underwent a comprehensive, ten-year restoration project. The plan to install underfloor heating cables, as well as a new floor, initiated a rescue excavation project with the aim of documenting and recovering any threatened archaeological features or material beneath the existing floor (Ødeby *et al.*, 2022). Although emphasis was on identifying pre-cathedral activity on the site, 77 medieval and post-medieval graves and 48 in-situ skeletons were uncovered. Preservation of the skeletons was found to vary substantially, with near-empty coffins/graves found directly on top of well-preserved skeletons. No graves from the pre-cathedral cemetery, initially identified in the 1960s, or remains of an earlier church were identified. The excavation and documentation work were challenging due to safety issues concerning the foundations of the building and the floor. Only small areas could be excavated at a time, and only to a certain depth (Ødeby *et al.*, 2022).

###### The material

Twelve individuals recovered during the 2021 excavation in the nave are included in the project. These display highly variable preservation conditions and degrees of completeness. Five of the skeletons sampled displayed an unusual degradation pattern noted in several graves during the nave excavation. A large proportion of the skeletons had disintegrated into a white powder, while other parts, often the lower legs, were well preserved.

Skeleton SZ2253/S14393.72 was well preserved but had been disturbed by a later burial (SA2264), thus only the skull, mandible, and a couple of vertebrae remained. The skull was covered by a fine layer of mortar and seems to be that of an adult male. The material was radiocarbon dated to AD 1219–1278 (95,4%) (Ødeby et al. 2022). This is one of the few confirmed medieval burials in the church and one of the few individuals from the High Medieval Period in Stavanger. The radiocarbon dating work of the Future Past project led to the identification of another skeleton, S143939.76, as high medieval. Skeleton SZ2617/S14393.76 was poorly preserved, and due to later disturbances, only the torso and hips survive. The skeleton was represented by ribs, vertebrae, clavicle, sacrum and hip bones, but these were fragmented. The skeleton seemed to have belonged to that of an adult female. The partial skull of an infant lying in the woman’s hip region suggests she was pregnant when she died. The remaining graves are assumed to be of post-medieval data (younger than 1537) based on stratigraphy and artefacts found.

##### The eastern trench 2022

During trenching for new pipes and cables, the faint trace of a possible grave cut was identified 3-5 meters away from the eastern facade of the cathedral. The Future Past research project received permission from the Norwegian cultural heritage authorities to investigate this, which turned out to be of early medieval date. The burial cut through cultural layers, including one interpreted as a cultivation layer, and into sterile subsoil. Unfortunately, the closeness of this trench to the cathedral wall, and the depth of more than 1 meter meant that the project did not get permission to extend the trench further to excavate the whole grave. Only the bones from the hip down were recovered. The alignment with the cathedral and the radiocarbon date range suggest it is an early burial, but one made after the cathedral was constructed.

##### The northern trench 2023: Future Past research excavation

Between February and April 2023, the Future Past project carried out a research excavation in the Stavanger cathedral cemetery. A 30 m^2^ trench, 6x5 meters, 1.5 meters from the northern wall of the choir, was investigated to a depth of 1-1.5 meters. This was considered an area with high potential to uncover intact medieval cultural layers, including the early cemetery, based on the 1967 finds (Rolfsen, 1968), knowledge from previous excavations and trenching in the cathedral area over the years (Fyllingen and Brun, 2017), and on a georadar investigation carried out in 2018 (Nau and Kristiansen, 2019). In total, 33 *in-situ* skeletons were recovered, along with 9 reburied coffins with skeletons and many loose/mixed skeletal remains. Around 600 samples of soil associated with the burials were for analyses of pollen, plant macrofossils, insect remains, lipids, and soil elemental chemistry.

The proximity to the cathedral choir wall, the foundations of which were ca 1.4 meters deep, meant that the whole area could not be opened at once. The first 60 cm were uncovered across the whole trench. This did not reveal any undisturbed burials. The trench was then divided into two trenches of 15 m^2^ each. The northern trench was excavated first. Here, a 19th-century trench with two pipes was uncovered, underneath which lay the nine redeposited coffins, oriented north-south, and placed on top of each other in two layers, head to toe. These were excavated in two phases, as they stretched between the northern and southern trenches. The coffins resembled those found inside the nave and are interpreted as coffins from the burial chambers underneath the floor that were emptied and dismantled during the 19th-century restoration project. The northern trench had to be refilled before the excavation of the southern trench could commence. In the southern trench, the first in-situ burial appeared at 60 cm. This top layer was defined as level I, consisting of 23 assumed post-medieval burials, found in heavily used and mixed cemetery soil. Grave cuts could not be distinguished, and many skeletons were cut by later graves or continued into the profiles of the trench. At the bottom of the trench, cut into the sterile sub-soil with clear grave cuts; ten burials were uncovered, later confirmed to be of medieval date. This was defined as level II.

All burials are assumed to be coffin burials, based on finds of many iron coffin nails, but no wood has survived except in the case of the redeposited coffins, where it was heavily degraded (see Fig. S1). The skeletons from these coffins were in perfect anatomical position, which means that they must have had some soft tissue intact, holding the skeleton together, when they were moved in the 19^th^ century. There was no soft tissue remaining, however, except for a couple of cases of remains of hair and/or wigs, and possible adipocere. It is assumed that the environment in the burial chambers was dry, leading to partial mummification by natural desiccation. In general, few finds were made in the northern trench apart from bones and coffin nails. At the bottom of the trench, one post-hole was discovered which was the only other structure found except for graves.

Before back-filling the southern trench, equipment for environmental monitoring was installed. Sensors logging environmental parameters; pH, oxygen levels, moisture, redox potential and temperature were installed into different layers and features. These will log data for at least five years since installation in early May 2023.

###### The material

Of the total 42 skeletons recovered, 23 were selected for further analyses as part of the Future Past project. All 10 medieval skeletons were included, 3 of the redeposited skeletons, and 10 from the post-medieval level I. Several are incomplete due to disturbances, and because parts of the skeletons extended beyond the boundaries of the trench. The sub-collection includes young and old individuals, and material of variable levels of preservation.

## Ogna

##### General background

The stone church at Ogna was built around 1300. This is based on the style of the west portal, an octagonal baptismal font, and a previous eastern window. The first mention of the church in historic records is in 1442. Both Sola and Ogna churches functioned as sea mark, or navigation mark for boats and ships, and many sailors gave generous gifts to the church. Around 1521, the church at Varhaug was the main church for the area, while Ogna was a local chapel. Around 1620, it was subsidiary to the church in Egersund. The cemetery has been expanded several times since 1856 in a western direction so that by 1950 it reached the main road (Lexow, 1958; Brendalsmo and Eriksson, 2016). Ogna was likely built by the same builders as of the bishop’s chapel in Stavanger and is the youngest and southernmost of the Rogaland medieval stone churches. After its completion, no new churches were built in the county for the next 300 years, due to economic and cultural stagnation, likely caused by factors such as plague, changes in trade routes, and changes in climate (Lexow, 1958).

##### Excavation and site description

The three individuals included in our study were uncovered in 1994 during work following a fire in 1991 (Karlberg, 1994). The church was repaired and rebuilt, and various trenches for pipes were dug around the building. An archaeologist was monitoring the work, and in one of the trenches (trench 2), ten intact graves were found. The trench was located east of the medieval church, from the north-eastern corner of the choir and outwards towards the churchyard fence. All graves were documented, and five were selected to be excavated. The graves were tentatively interpreted as medieval due to the position of the skeleton, direction of the grave, burial depth and the presence of hand-made coffin nails. The report states that rough weather conditions, failing equipment and the haste involved limited the quality of the documentation. The ten graves were found across a 23 m long, 1.2 m wide trench, at ca 0,9 m. The burial conditions were described as follows: *Underneath asphalt and gravel, a mixed layer with pebbles, then a darker brown sand-mixed layer and finally light greyish-brown shell sand/marine sand*. The burials were cut into the layer of marine sand. Clear zones of mineral precipitates between the layers testify to the stability of the soil conditions. The graves did not cut into any older graves and were not cut by others, and there were little mixed skeletal remains in the surrounding soil. This is unusual in a medieval cemetery, as these are often intensely used. This suggests that this period of the churchyard was only used for a limited time, and that they were clearly marked and possibly buried within a relatively short time span (Karlberg, 1994).

##### The material

Three of the five individuals excavated are included in our study: Skeletons no 2, 3 and 5 from graves 2, 4 and 8 (museum numbers S12124.2, 3 and 5).

For grave 2, remains of coffin wood and iron rivets were found. Almost the whole grave cut was intact within the trench, and except for the foot bones, the entire skeleton is present. The individual is an older female (age range 60-80). She was suffering from severe osteoarthritis. She had lost most of her teeth quite some time before she died, and her remaining teeth were heavily worn. The skeleton in burial 4 was that of a man, aged 30-50. He had a healed fracture in the right tibia. Only the lower half, from the hips down, was available for excavation within the trench. Burial 8 contained no coffin remains. The skeleton was poorly preserved, and only skull fragments and ribs were found. This was tentatively interpreted as a female, 30-50 years of age (Karlberg, 1994; Sellevold, 2004).

At the time of excavation, these graves were assumed to be medieval. However, radiocarbon dating carried out by the Future Past project has proven otherwise, as they show that the skeletons are no older than the 1600s and have thus been classified as young/post-medieval.

## Utstein

#### General Background

Like Avaldsnes, Utstein is strategically situated, by the main sea route, and between Stavanger and the sound by Avaldsnes, on an island just north of the city. The bay by the abbey was a safe haven for ships for centuries, before entering rougher seas in fjords or out at sea. In early medieval times, a large farm was situated in the bay. The strategic location meant one could control not only the main sea route along the coast, but also the network of fjords constituting a route to the inland. The estate included good soils for cultivation and extensive land for grazing. All of this provided a strong basis for a regional power centre. In the saga literature, Utstein is described as one of the royal manors where Harald Fairhair (ca 875 – c 932) stayed in his old age (Lexow, 1963). Utstein was one of four residences of the king, and it is situated not far from Hafrsfjord, where Harald, according to a skaldic poem, won a battle that led to the first unification of Norway into one kingdom (Haug, 2014).

The foundation year of the abbey is not certain. It is first mentioned in written sources in 1280, in a testament where a gift is given to the abbey. The traditional view is that its foundation relates to the closure of the St Olav’s monastery in Stavanger, as the St Olav’s estate seems to have been transferred to Utstein (Lexow, 1963). The Utstein Abbey belonged to the Augustinian order, and its patron saint was St. Lawrence. The St. Olav monastery in Stavanger belonged to the Benedictine order and was likely connected to the establishment of the cathedral and the bishopric in Stavanger. The transfer of monasteries from the Benedictines to the Augustinians after 1250 was a European-wide phenomenon. The baptismal font at the abbey is stylistically dated to the 1150s and thus may have originally belonged to the St Olav’s monastery, as there is, according to Lexow, little evidence that there was a church at Utstein in the 1100s (Lexow, 1963). Lexow (ibid.) suggests that the estate was founded by Magnus Lagabøte (the lawmaker) in the 1260s, initially as a royal farm and only later converting to an abbey. More recently, Haug (2014) has proposed an earlier date of founding, suggesting that the abbey was established in the same period as the other Augustinian convents, namely in the second half of the 12^th^ century.

In 1515, there was a conflict between the abbot at Utstein and bishop Hoskold in Stavanger. The bishop’s men first raided the abbey while the abbot was in Denmark, taking with them both food, treasures and cattle. A second time, the bishop came with his men and broke in to arrest the abbot, who hid in the tower and avoided capture. Three days later, the bishops’ men came to lay an interdict on the abbey, prohibiting the abbey from carrying out its religious services. In a final attack led by the bishop, the bishop and his men came with catapults, slings and ladders and caught the abbot in his bed. With the abbot in prison, Hoskold went back to raid the abbey (Lexow, 1963). In the following years, the abbey was in a poor state. In 1530, the abbot Trugels Amundsøn had to sell one of the farms in Stavanger, testifying to the abbey’s deep poverty. There were also local conflicts in this period regarding who should reign at Utstein. One of Norway’s most powerful men at the time, Vincent Lunge, attempted to take over the abbey in 1530, capturing the abbot and putting his own bailiff in charge, but Bishop Hoskold later reinstated his abbot. Upon the abbot’s death, Vincent Lunge again tried, unsuccessfully, to seize the abbey, but Hoskolds abbot, Jørgen Hanssøn, remained in power until the abbey’s dissolution in 1537. Hanssøn had to travel to Bergen to hand in the abbey’s silver, and that marked the end of monastic life at Utstein (Lexow, 1963).

After its dissolution, the Danish crown took over the estate of the abbey, and in the period until 1665, it was deeded to nine Danish noblemen. Only three of them are known to have visited the estate, and bailiffs were appointed to see to the daily management of the estate, including collecting fees from the associated farms. Several of these bailiffs are known from historical records, often due to conflicts and misdemeanours, including fraud, alcohol misuse and violence. The first of these was Trond Ivarssøn, who took over on the condition that he tend to the welfare of the monks (Lexow, 1963).

In 1539, the abbey was again raided, this time by the pirate Christoffer Trondsson Rustung. Archaeological evidence uncovered in the 1950s suggests that the abbey was burned during the raid. It seems that the abbey was abandoned after this. The first documented ‘rental agreement’ is from 1547, and the first documented reoccupation and use of the church is from 1594. The bailiff Knud Knudssøn makes an agreement with the priest at Rennesøy, which includes him funding a priest at the church so that the local population get the opportunity to attend church more frequently. Afterwards, the church would revert to the Hauskjen parish (Lexow, 1963).

#### The excavations and the material

Only three individuals uncovered from underneath the church floor are included in Future Past. Two were uncovered in the 1930s during excavations by Harald Hals, conservator at Stavanger Museum. Although Hals’ diaries mention finds of skeletal remains, they provide little detail about these. An exception is the description of a stone-set grave in the nave. This was partially collapsed, and filled with gravel, containing a few post-cranial elements and the cranium of, according to the Anatomical Institute in Oslo, a man aged 40-45 years. In the nave, Hals describes the foundations of the church walls, where human bones incorporated into the wall make him suspect that it disturbed an earlier burial ground. In the choir, a wall that seems to predate the building of the monastery is found. Outside of this wall, he observed ‘several’ skeletons, all oriented ‘correctly’ (Hals, 1934).

The third individual was excavated by Dorothea Fischer in 1964. Dorothea was the wife of architect Gerhard Fischer, a Norwegian architectural historian involved in restoration works both in Stavanger and at Utstein. Records for the excavations are extremely limited. After excavation, the remains were sent to the Anatomical Institute, the University of Oslo. The letters between Stavanger Museum and the Anatomical Institute are so far the only written records available regarding these specific remains.

Future Past’s radiocarbon dating of the remains shows that they span the entire history of the abbey, from the foundation period during early medieval times, through the high medieval to late medieval/post-medieval, when the abbey went out of use. The skeletal material was stored at the University of Oslo until 2022, which, since excavation, has been kept in Oslo but was transferred to the Museum of Archaeology in 2022, in connection with the Future Past project.

Individual A4105, represented by the skull only, has the least amount of associated contextual information. Presumably, it was excavated by Hals during his work at the abbey in 1934 and was most likely found in the nave, as the dates of his diary suggest that the nave (Hals, 1934). Interestingly, this is a female, and it is so far radiocarbon dated to the early medieval, suggesting it could stem from the period before the establishment of the monastery, when it was still a royal manor, or support an earlier period of construction.

Individual A4996 is described in a 1964 letter from Stavanger Museum to the Anatomical Institute (Lexow, 1964). The remains, consisting of both cranium and post-cranial elements, were found during Fischer’s 1964 excavation underneath the floor of the nave. It was found together with another skeleton, on top of the foundations of a building pre-dating the church, thus according to Lexow, younger than AD 1265. Lexow suggests that this building could have been a chapel on the royal farm. There was no sign of coffins, and Lexow assumes they were monks who, by tradition, were buried in their cloaks only. Bernhard Getz at the Anatomical Institute sent a brief reply stating that the remains were those of a robust male, 45-55 years of age, the estimated height and shape of skull, and that there was little else to report on the skeleton (Getz, 1964). Our radiocarbon dates prove Lexow right in this assumption, with the 95,4% probability span being 1296 to 1394. This includes the period of the mischievous abbot Eirik who in 1333 was accused of, among other things, letting his sister sing together with the monks, publicly revealing the secret testimonies of two monks, and removing valuables from the abbey (Lexow, 1963).

Individual A4114 is also represented by a cranium only. The letter from Hals to Schreiner at the Anatomical Institute in Oslo, dated 1935, describes that it was taken from underneath the church floor and, according to its stratigraphical position, should be at least 500 years old (Hals, 1935). According to Hals, it was a coincidental discovery during other work and had to be removed due to it being exposed and in the way. He could see that the rest of the skeleton was lying *in situ*, but refrained from collecting it both due to being less accessible and due to the troubles he had experienced during the excavations of graves the year before. In the letter, Hals explains that he marked the spot where the skull had been taken from clearly. He also notes in the letter that the skull is an interesting case due to its size and a wound, which seems to be a healed sword cut, in the forehead. Despite Hals’ assumption of a Medieval date, Future Past’s radiocarbon dating results suggest that it is no more than 500 years old. The time range of the date covers the late medieval to about a hundred years into the post-medieval period, which was a tumultuous part of the abbey’s history as outlined above. The individual could be a monk wounded in the conflict with the Stavanger bishop in 1515, or an inhabitant of the abbey defending it against the pirate Rustung in 1539.

## Hausken

### General background

The medieval church at Hausken, located on Rennesøy, an island north of Stavanger, is first mentioned in written records in 1327. 17^th^ and 18th-century descriptions refer to it as a stave church. This building was partially replaced by a timber-built church in 1752, which was subsequently demolished and replaced by the current wooden church in 1857 (Brendalsmo and Eriksson, 2016). There are a few finds from the medieval period, but parts of a baptismal font, an altar and a tombstone with runic inscriptions have been saved (Lorvik, 2023).

### The site/excavation

In 2008, archaeological monitoring was required due to the installation of a new drainage system around the current church (Lorvik, 2023). A total of 63 burials were recorded, most of which were disturbed by later burials. The skeletal remains were in a poor state, and only 25 graves contained preserved material, but preservation levels also varied across the different trenches (material from trench E was better preserved than that from trench A). Stratigraphically, at least 20 of the graves must be older than the church of 1857. No coffins were preserved, but traces of coffins and coffin nails were found in 20 of the graves. There were no other finds. The graves were shallow, 0.6-1 meters in depth, and partially cut into the porous phyllite bedrock. The project was carried out in the winter, leading to difficult working conditions (Lorvik, 2023).

### The material

Nine individuals from the 2008 excavation, from trenches A and E, have been included in the Future Past project. All samples were radiocarbon dated, resulting in a total age range of 1491 to 1800 AD, almost all most likely belonging to the post-medieval period, and confirming that they are older than the current church. Four dates centre around the 1500s, three around the 1600s and two around the 1700s. All skeletons are poorly preserved, with few and fragmented skeletal elements remaining, some with just one skeletal element, like the arm bone from grave 41 (S14604.12).

## A note on the dating of the material

Over half of the skeletal collection used for this research has been radiocarbon dated. Remains, which, based on archaeological context, were highly likely to be post-medieval in date, were not dated. This was due to the lack of precision in the radiocarbon dating of post-medieval/early modern remains. Furthermore, the radiocarbon dates obtained have not yet been corrected for the marine reservoir effect based on stable isotope analyses, nor modelled based on stratigraphy and other archaeological information that may help constrain the results. This is a work in progress, and the results will be published soon. This may shift some dates somewhat, but in most cases, it is not expected to change the interpretation of the archaeological period. For the current study, it was considered sufficient to use the general period assignment, medieval or post-medieval (old/recent).

### Bibliography

Barrett, G.T., Ebert, B. and Allen, K. (2025) ‘Radiocarbon mortar dating of newly uncovered medieval ceiling paintings at Stavanger Cathedral, Norway’, *Journal of Cultural Heritage*, 71, pp. 402–411. Available at: https://doi.org/10.1016/j.culher.2024.12.017.

Bauer, E.L. (2017) ‘14. The High Medieval Royal Manor’, in D. Skre (ed.) *Avaldsnes - A Sea-Kings’ Manor in First-Millennium Western Scandinavia*. De Gruyter, pp. 277–308. Available at: https://www.degruyterbrill.com/document/doi/10.1515/9783110421088-016/html (Accessed: 12 June 2025).

Bergland, T. (2021) Bergland, T., 2021. ‘Osteologisk analyse av skjeletter fra Avaldsnes Middelalderruin’ (No. 21). Norsk institutt for kulturminneforskning (NIKU).

Brendalsmo, J. and Eriksson, J.-E.G. (2016) ‘Kildegjennomgang. Middelalderske kirkesteder i Rogaland Fylke.’ Riksantikvaren. Available at: http://hdl.handle.net/11250/2424650.

Brendalsmo, J. and Paasche, K. (2017) ‘Stavanger – før det ble en by’, *Historisk tidsskrift*, 96(02), pp. 103–123.

Daasvand, T. (2012) *Formuende, middelmådige og fattige : den sosiale sammensetningen i Stavanger 1661 - 1801*. Universitetet i Stavanger: Arkeologisk museum. Available at: https://uis.brage.unit.no/uis-xmlui/handle/11250/181486 (Accessed: 2 July 2025).

Denham, S.D. (2014) ‘Commingled remains from Stavanger Cathedral’, in B.J. Sellevold (ed.) *Old Bones – Osteoarchaeology in Norway: Yesterday, Today and Tomorrow.* Oslo: Novus Forlag, pp. 117–136.

Ebert, B. (2024) ‘Learning from the Past: Rediscovering Traditional Medieval Wood Tar Adhesives for Sustainable Stone Conservation and Built Heritage’, *Studies in Conservation*, 69(sup1), pp. 63–71. Available at: <https://doi.org/10.1080/00393630.2024.2339728>.

Ebert, B., Bjelland, T., Philippsen, B., 2025. Hidden Structures: Medieval Wooden Scaffolding Reveals New Evidence for the Dating and Construction of Stavanger Cathedral in Norway. International Journal of Wood Culture 5, 167–199. <https://doi.org/10.1163/27723194-bja10050>

Ekroll, Ø. (2013) ‘Fjernsyn - nærsyn - vidsyn: Stavanger sett på avstand’, *Stavangeren*, 3, pp. 43–52.

Ersland, G.A. (2013) ‘Mellomalderbyen Stavanger’, *Stavangeren*, 3, pp. 33–42.

Fyllingen, H. and Brun, W. (2017) *Arkeologisk undersøkelser i og rundt Stavanger domkirke*. Stavanger: Arkeologisk Museum UiS.

Getz, B. (1964, December 8) [Letter to Stavanger Museum]. The Museum of Stavanger archives (Muségaten 16, post code 4010), Stavanger, Norway.

Hals, H. (1934) [Diary of restoration and excavation work at Utstein abbey]. The Museum of Stavanger archives (Muségaten 16, post code 4010, Åmøy repository archives, Folder St.K a 061 ‘Utstein Kloster Dagbok, avskrifter, korrespondanse 1380-1965), Stavanger, Norway.

Hals, H. (1935, August 26) [Letter to Professor Schreiner]. The archives of the Biological Anthropological Collection at the University of Oslo (Domus Medica, Gaustad,
Sognsvannsveien 9, post code 0372), Oslo, Norway.

Haug, E. (2014) ‘Augustinian canons and benedictine monks in the Medieval Stavanger diocese’, in L. Bisgaard et al. (eds) *Monastic culture: The long thirteenth century: Essays in honour of Brian Patrick MacGuire.* Denmark: University Press of Southern Denmark.

Helle, K. (1999) ‘Olavskirken 1250-1350 - sognekirke og kongelig kapell’, in S.I. Langhelle and B. Lindanger (eds) *Kongskyrkje ved Nordvegen. Olavskyrkja på Avaldsnes 750 år.* Lokahistorisk stiftelse.

Høgestøl, M. and Sandvik, P.U. (2020) ‘Skjeletta frå Stavanger domkyrkje – ei lang og innfløkt soge’, *Viking*, 83(1), pp. 157–178.

Hommedal, A.T. and Sellevold, B.J. (1999) ‘Skal førast til kyrkja og gravast i heilag jord - om gravene og skjelettfunna frå mellomalderkyrkja på Sola’, *Haug ok Heidni*, (2), pp. 10–15.

Hommedal, A.T. and Sellevold, B.J. (2017) ‘Fra jord til arkiv. Om dokumentasjon av arkeologiske gravfunn fra middelalder og nyere tid’, in G. Lillehammer and L. Selsing (eds) *Jenny-Rita.org. Et utradisjonelt skrivested i Jenny Ritas ånd.* Stavanger, Norway: Arkeologisk Museum, Universitetet i Stavanger (AM Profil), pp. 58–68.

Johansen, L.O. (2005) *Arkeologiske undersøkelser i Byparken og rundt Domkirken, Eiganes gnr. 58, Stavanger kommune 2005*. 5. Arkeologisk Museum i Stavanger.

Karlberg, I. (1994) ‘Ogna Kirke Hå kommune, Rogaland fylke. Nyreising av kirke etter brann. Rapport fra arkeologisk overvåkning 7. desember 1994’. Riksantikvaren.

Lexow, J.H. (1958) *Middelalderens steinkirker i Rogaland*. Aktietrykkeriet i Stavanger. Available at: https://www.nb.no/items/11cc8ac7dfe47f1591210fea699c2e15?page=0&searchText=middelalderens%20steinkirker (Accessed: 1 July 2025).

Lexow, J.H. (1963) *Utstein kloster etter reformasjonen*. Stavanger: Dreyer Aksjeselskap.

Lexow, J.H. (1964, October 22) [Letter to the Anatomical Institute]. The archives of the Biological Anthropological Collection at the University of Oslo (Domus Medica, Gaustad,
Sognsvannsveien 9, post code 0372), Oslo, Norway. Lorvik, K. (2023) *Hausken kirke, Rennesøy kommune, Rogaland. Arkeologisk undersøkelse og humanosteologisk analyse*. 224. Oslo: NIKU.

Næss, J.R. and Sellevold, B.J. (1990) ‘Graver fra historisk tid: vitenskapelig kilde og forvaltningsproblem. Med spesiell vekt på “kristne graver” fra middelalderen.’, in O.J. Benediktow (ed.) *Collegium Medievale. Forening for middelalderforskere.* Oslo.

Nau, E. and Kristiansen, M. (2019) *Stavanger Domkirke. Georadarundersøkelse ved Domkirken og på Domkirkeplassen, Stavanger, Rogaland.*

Nordlie, E. and Ødeby, K. (2021) ‘Arkeologiske undersøkelser ifm. konservering av middelalderruin’, *242* [Preprint]. Available at: https://niku.brage.unit.no/niku-xmlui/handle/11250/2822986 (Accessed: 12 June 2025).

Nordlie, E. and Sand-Eriksen, A. (2019) ‘Middelalderruin. Avaldsnes Prestegård, 86/1, Karmøy kommune, Rogaland.’ Available at: https://www.duo.uio.no/handle/10852/67362 (Accessed: 12 June 2025).

Rolfsen, P. (1968) ‘Rapport fra utgravningen i Stavanger domkirke’.

Rolfsen, P. (2021) ‘Stavanger domkirke - en arkeologisk utgravning under koret’, *Viking*, LXXXVI, pp. 139–170.

Sellevold, B.J. (1996) ‘Kvinnen, kirken og døden: Middelalderbegravelser i Norden’, in N. Damsholt, G. Jacobsen, and N.H. Homqvist-Larsen (eds) *Kirkehistorier. Rapport fra et middelaldersymposium.* København: Museum Tusculanums Forlag.

Sellevold, B.J. (2004) *Ogna kirke, Hå k., Rogaland, S12124. Undersøkelse av menneskeben fra en middelalderkirkegård*. 1. NIKU.

Sellevold, B.J. (2006) *Skjelettfunn fra Byparken, Eiganes gnr 58, Stavanger k., Rogaland*. 1. NIKU.

Sellevold, B.J. and Hommedal, A.T. (2008) ‘Archaeological Bones – The Anatomy of an Abandoned Churchyard’, *Norwegian Archaeological Review*, 41(1), pp. 71–84. Available at: https://doi.org/10.1080/00293650802075513.

van der Sluis, L. (2012) *A view into the lives of early Christians: palaeodietary investigation of a mulitperiod churchyard in Stavanger, Norway, using stable isotopes (C, N, H, S) of bone collagen*. 08. Amsterdam: VU University, p. 56.

Stige, M. (2013) ‘Stavanger domkirke - den menneskelige katedralen’, *Stavangeren*, 3, pp. 53–64.

Stylegar, F.-A. *et al.* (2011) ‘To ansiktsmasker og en ring fra Avaldsnes i Rogaland : ett svar og flere spørsmål’, *Fornvännen*, 106(1), pp. 8–25.

Utne, B.S. (1988) ‘Byen og kirken’, in B. Wold Johnsen et al. (eds) *Stavanger Domkirke i sentrum*. Stavanger: Redaksjonskomiteen, pp. 9–36.

Ødeby, K. *et al.* (2022) ‘Arkeologisk undersøkelser i krypkjelleren, Stavanger Domkirke. Stavanger domkirke, Stavanger kommune, Rogaland’, *465* [Preprint]. Available at: https://niku.brage.unit.no/niku-xmlui/handle/11250/3014177 (Accessed: 20 June 2025).
