# Supplementary figures and images for: Histological and metagenomic analysis of microbial communities in archaeological human bones
Source: PLoS One. 2026 May 27;21(5):e0340244. doi: 10.1371/journal.pone.0340244 (PMC13215491; doi:10.1371/journal.pone.0340244)

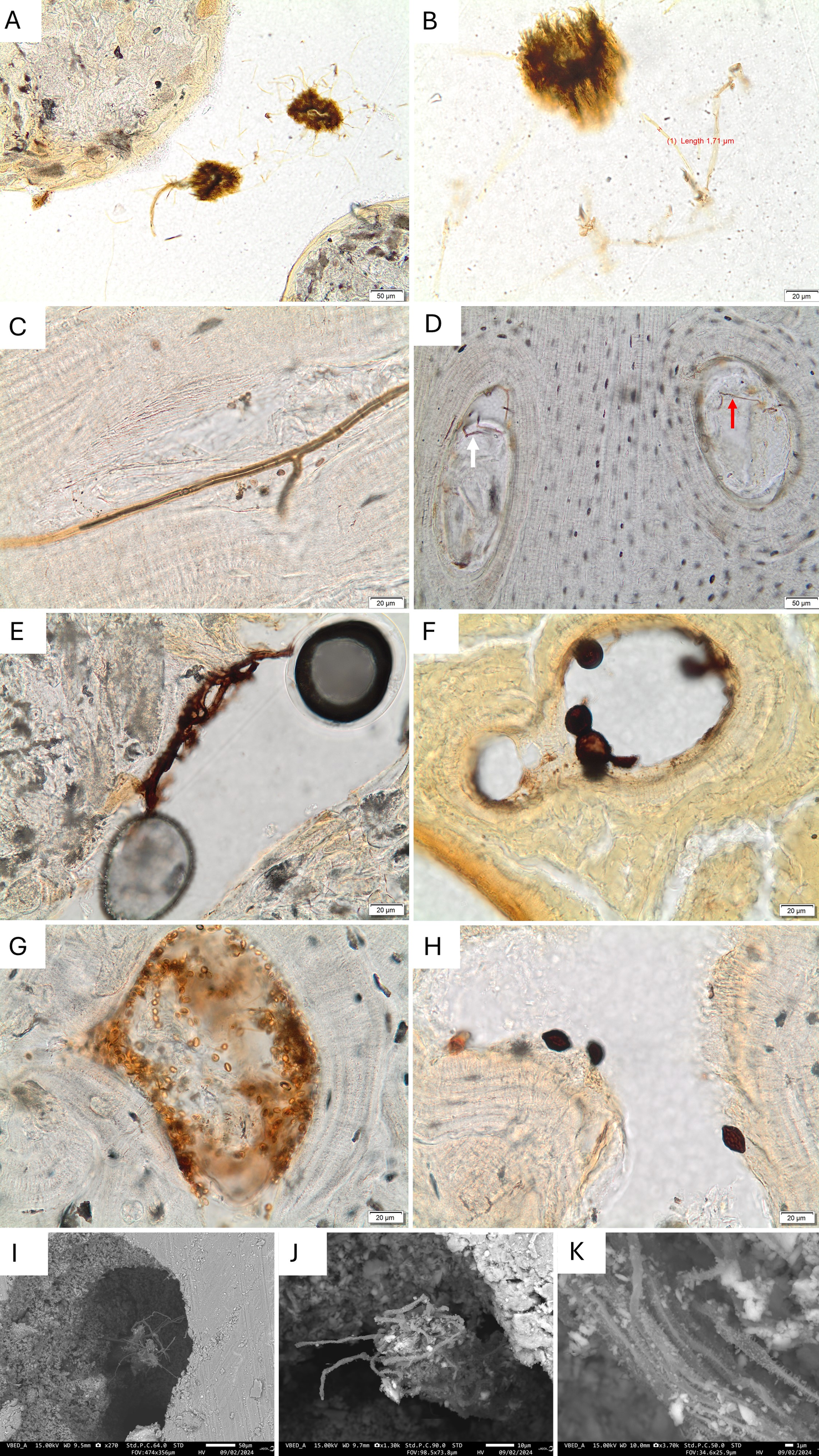

Supplement: S1 Fig — (A) Fine hyphae and bundles of hyphae within the trabecular bone of S14393.83. (B) Detail of the fine hyphae and bundles in the same sample as in (A). (C) Thicker hyphae in sample S14393.72, clearly branching and segmented. The brown grains seen along the hyphae could be spores, or the cross-section of similar hyphae. (D) Fine hyphae (arrows) within two Haversian canals (blood channels) of sample S14393.118. (E) Fungal hyphae in a Haversian canal in S10294.18. The two large spheres are air bubbles in the embedding resin. (F) Spherical spores or sporangia within a Haversian canal in SZ20610. (G) A mass of almond-shaped spores filling a Haversian canal in SZ21164. (H) Lemon-shaped fungal spores in S14393.72. (I-K) SEM-images in backscatter mode of fine fungal hyphae, in pores of an unpolished bone sample of SZ20839. The diameter is roughly one µm and the hyphae have a hairy surface. (TIF) [file pone.0340244.s002.tif]

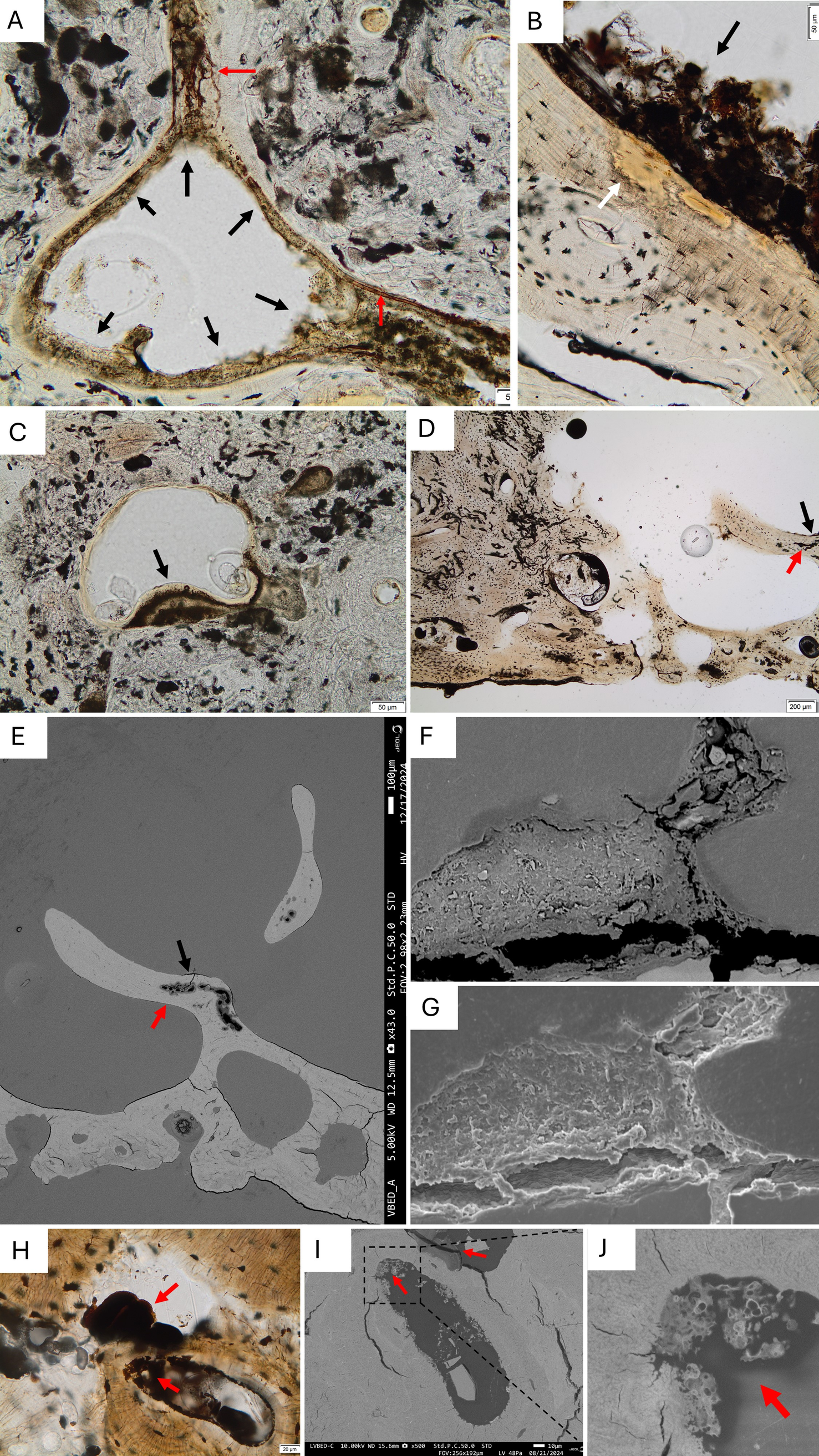

Supplement: S2 Fig — (A) A grainy layer (black arrows) coating the surface of a Haversian canal and partially filling branching channels in SZ20610. Fungal hyphae are also present (arrows). (B) A grainy layer (black arrow) on the outer surface of A5133. Note the yellow etched surface area directly underneath (white arrow). (C) A grainy layered dome (black arrow) on the surface of a Haversian canal in SZ20711. (D) Similar grainy surface deposits were seen in the light microscope on sample STVA5132. Such a desposit (black arrow), and destructive foci in the bone underneath (red arrows), were studied further in the scanning electron microscope (E-G). (E) An SEM image of the same area as seen in the light micrsocope in (D), the arrows pointing to the same features. (F-G) SEM images of the surface deposit seen in (D) and (E) at higher magnification, in backscatter (F) and secondary electron mode (G). It has a dome-shape, consisting seemingly of a fibrous mass, and has low density. The large cracks in the bone and between the deposit and the bone surface is likely caused by the sample preparation and/or the vacuum in the electron microscope. (H-J) Possible biofilm observed in sample SZ20839, within Haversiancanals (red arrows), in light microscope (H), and in SEM (I-J), where J shows a magnification of one part, revealing a porous structure and a low-density material. No chemical analyses were carried out, but it is clear from the back scatter image that there are no concentrations of dense materials such as manganese and iron thus the dark reddish-brown color seen in the light microscope is likely caused by an organic component. (TIF) [file pone.0340244.s003.tif]

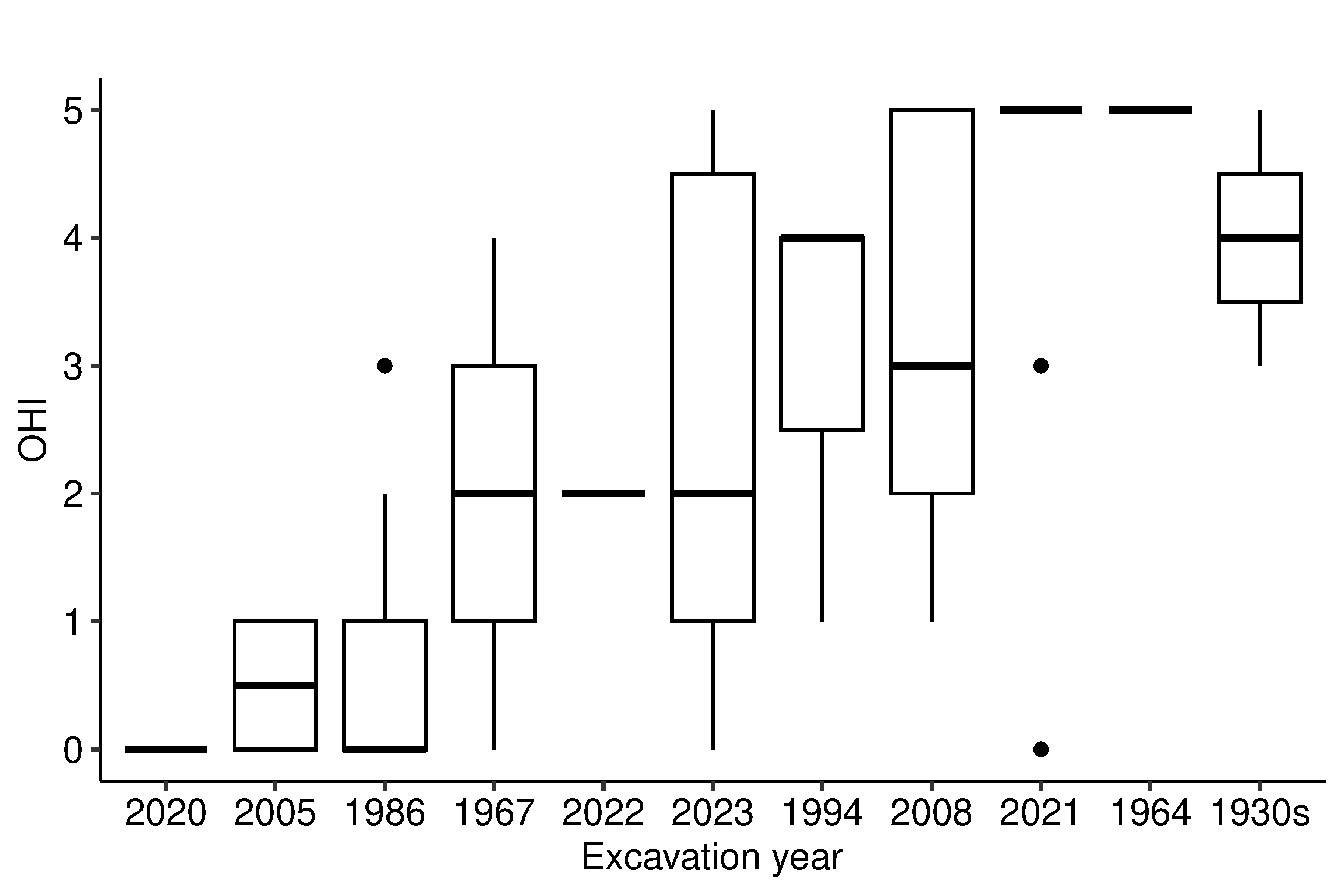

Supplement: S3 Fig — (TIF) [file pone.0340244.s004.tif]

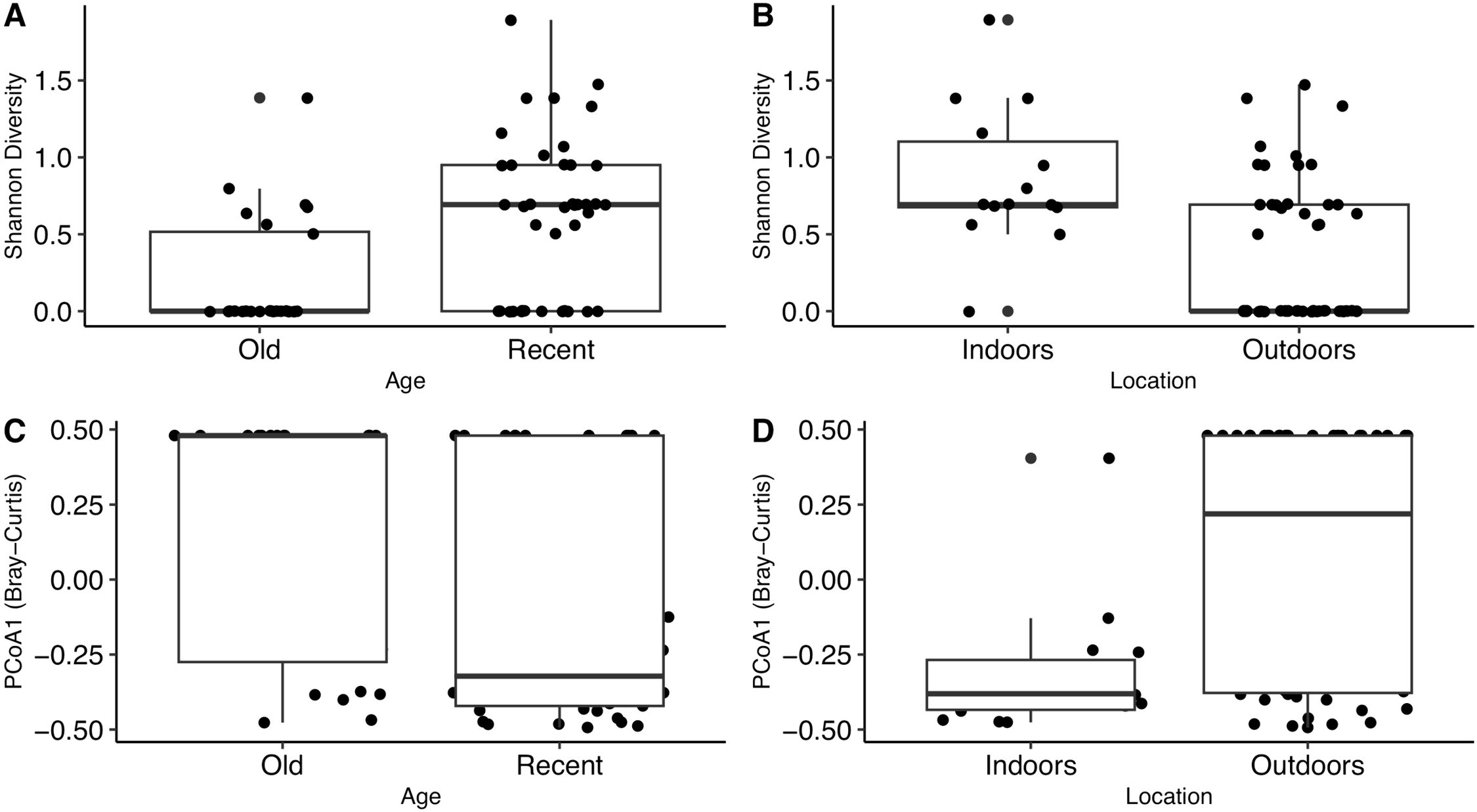

Supplement: S4 Fig — (A) Shannon diversity between old and recent samples. (B) Shannon diversity between indoor and outdoor samples. (C) Distribution of the first principal coordinate (PCoA1) derived from Bray–Curtis dissimilarities between old and recent samples. (D) Distribution of the first principal coordinate (PCoA1) derived from Bray–Curtis dissimilarities between indoor and outdoor samples. “Old” refers to periods prior to the post-medieval, while “recent” refers to post-medieval periods. Statistical tests: Wilcoxon rank-sum test for Shannon diversity (p < 0.005) and Kruskal–Wallis test for PCoA1 differences (p_age = 0.03; p_location = 0.003). (TIF) [file pone.0340244.s005.tif]

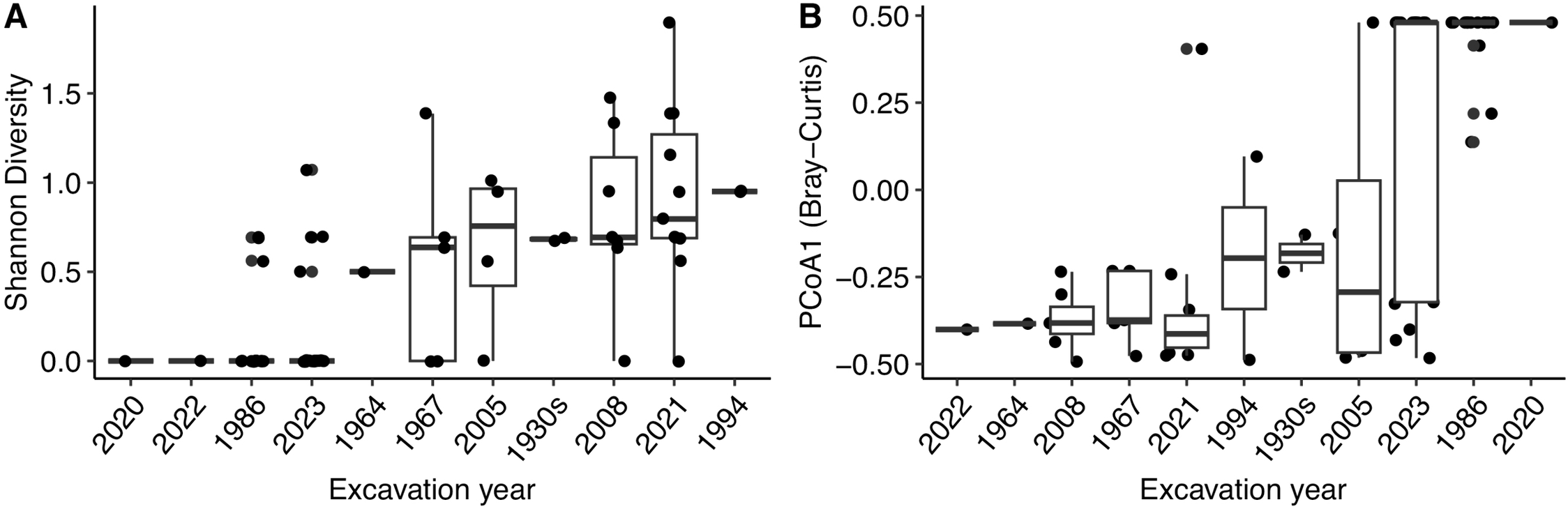

Supplement: S5 Fig — (A) Shannon diversity (B) Distribution of the first principal coordinate (PCoA1) derived from Bray–Curtis dissimilarities. (TIF) [file pone.0340244.s006.tif]

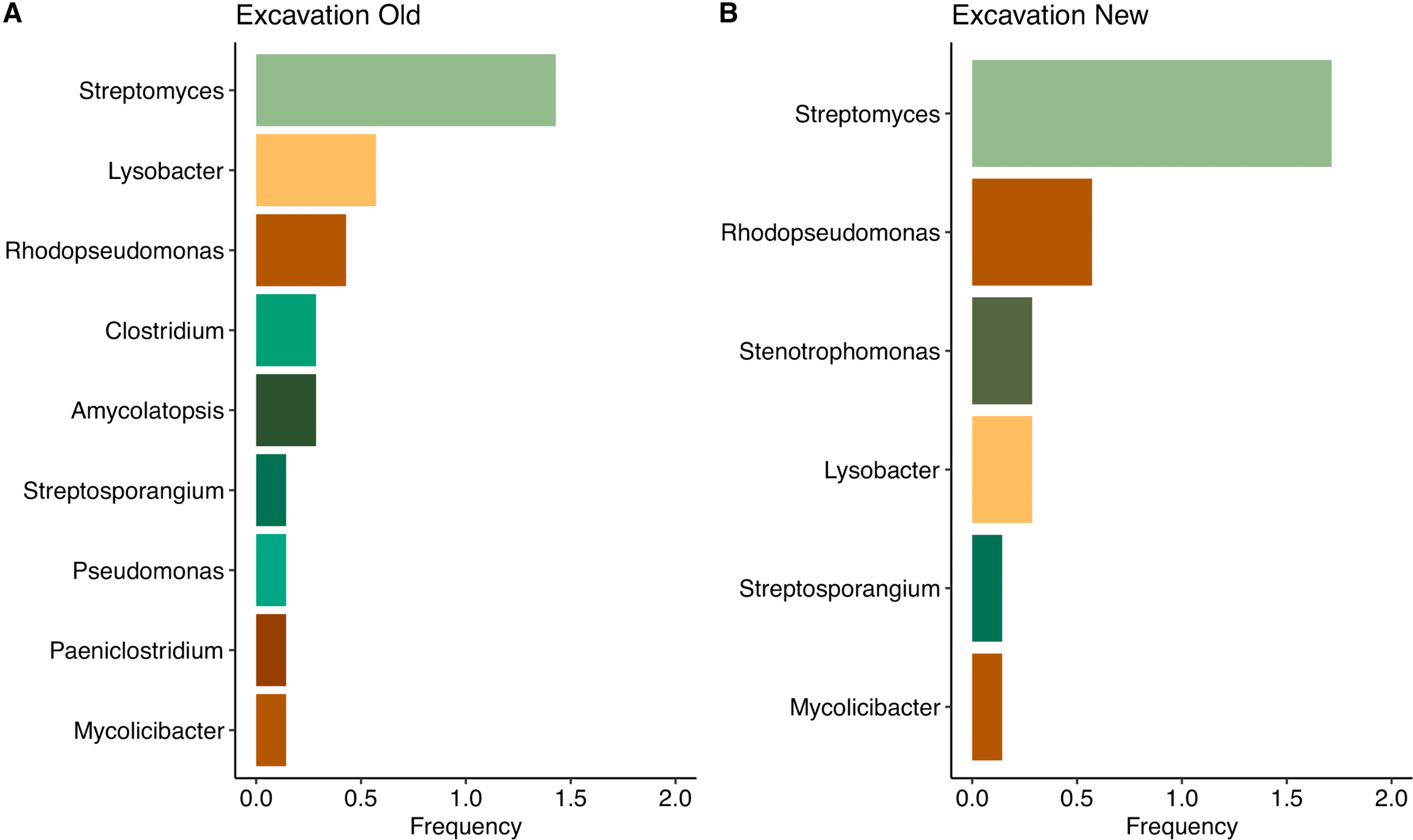

Supplement: S6 Fig — Stavanger North Cemetery samples with only OHI scores 4 and 5, grouped into (A) Excavation Old (1967, 1994, 2008 excavations, n = 7) and (B) Excavation New (2023 excavation, n = 7). (TIF) [file pone.0340244.s007.tif]

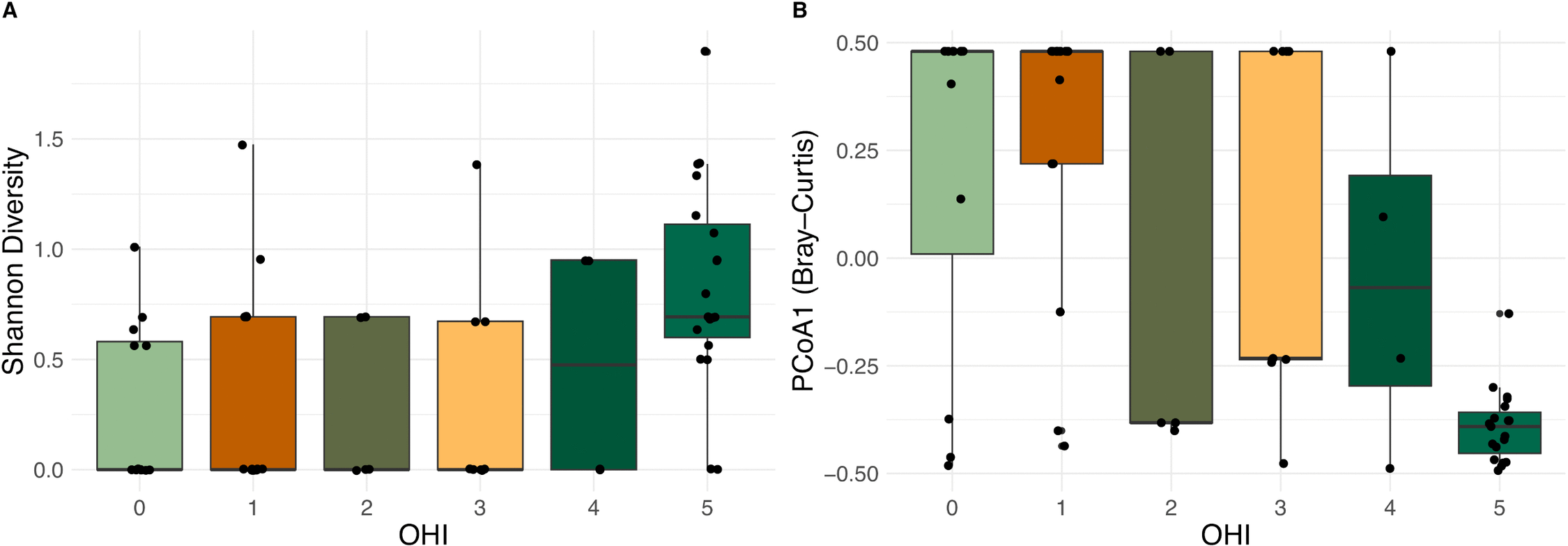

Supplement: S7 Fig — (A) Shannon diversity (B) Distribution of the first principal coordinate (PCoA1) derived from Bray–Curtis dissimilarities. (TIF) [file pone.0340244.s008.tif]
