# Supplementary material for: Histological and metagenomic analysis of microbial communities in archaeological human bones
Source: PLoS One. 2026 May 27;21(5):e0340244. doi: 10.1371/journal.pone.0340244 (PMC13215491; doi:10.1371/journal.pone.0340244)
Supplement: Attachment — Submitted filename: Response_to_Reviewers.docx [file pone.0340244.s015.docx]

Journal requirements:

When submitting your revision, we need you to address these additional requirements.

1. Please ensure that your manuscript meets PLOS ONE's style requirements, including those for file naming. The PLOS ONE style templates can be found at

https://journals.plos.org/plosone/s/file?id=wjVg/PLOSOne_formatting_sample_main_body.pdf and

<https://journals.plos.org/plosone/s/file?id=ba62/PLOSOne_formatting_sample_title_authors_affiliations.pdf>

**We have gone through the style requirements of PLOS ONE and prepared the manuscript and all files accordingly.**

2. When completing the data availability statement of the submission form, you indicated that you will make your data available on acceptance. We strongly recommend all authors decide on a data sharing plan before acceptance, as the process can be lengthy and hold up publication timelines. Please note that, though access restrictions are acceptable now, your entire data will need to be made freely accessible if your manuscript is accepted for publication. This policy applies to all data except where public deposition would breach compliance with the protocol approved by your research ethics board. If you are unable to adhere to our open data policy, please kindly revise your statement to explain your reasoning and we will seek the editor's input on an exemption. Please be assured that, once you have provided your new statement, the assessment of your exemption will not hold up the peer review process.

**We thank the editor for this clarification. The raw sequencing data have already been deposited in the European Nucleotide Archive (ENA) under project accession number PRJEB104929. We have updated the Data Availability Statement accordingly to clarify that the data are archived in a public repository and will be fully accessible upon publication.**

3. We note that Figure 1 in your submission contain [map/satellite] images which may be copyrighted. All PLOS content is published under the Creative Commons Attribution License (CC BY 4.0), which means that the manuscript, images, and Supporting Information files will be freely available online, and any third party is permitted to access, download, copy, distribute, and use these materials in any way, even commercially, with proper attribution. For these reasons, we cannot publish previously copyrighted maps or satellite images created using proprietary data, such as Google software (Google Maps, Street View, and Earth). For more information, see our copyright guidelines: http://journals.plos.org/plosone/s/licenses-and-copyright.

We require you to either (1) present written permission from the copyright holder to publish these figures specifically under the CC BY 4.0 license, or (2) remove the figures from your submission:

1. You may seek permission from the original copyright holder of Figure 1 to publish the content specifically under the CC BY 4.0 license.

We recommend that you contact the original copyright holder with the Content Permission Form (http://journals.plos.org/plosone/s/file?id=7c09/content-permission-form.pdf) and the following text:

“I request permission for the open-access journal PLOS ONE to publish XXX under the Creative Commons Attribution License (CCAL) CC BY 4.0 (http://creativecommons.org/licenses/by/4.0/). Please be aware that this license allows unrestricted use and distribution, even commercially, by third parties. Please reply and provide explicit written permission to publish XXX under a CC BY license and complete the attached form.”

Please upload the completed Content Permission Form or other proof of granted permissions as an "Other" file with your submission.

In the figure caption of the copyrighted figure, please include the following text: “Reprinted from [ref] under a CC BY license, with permission from [name of publisher], original copyright [original copyright year].”

**We thank the editor for raising this important point. Figure 1 does not contain any proprietary or copyrighted map or satellite imagery (e.g., Google Maps, Google Earth, or other commercial basemaps). The country boundaries were obtained from Natural Earth (public domain), and the Rogaland administrative boundaries (NUTS-2 region NO04) were obtained from Eurostat GISCO, both of which are openly available and compatible with CC BY 4.0 licensing requirements. The figure was generated entirely in R (ggplot2, sf) using vector boundary data. We have clarified the data sources in the figure caption to ensure full transparency and compliance with PLOS ONE’s licensing policy.**

2. If you are unable to obtain permission from the original copyright holder to publish these figures under the CC BY 4.0 license or if the copyright holder’s requirements are incompatible with the CC BY 4.0 license, please either i) remove the figure or ii) supply a replacement figure that complies with the CC BY 4.0 license. Please check copyright information on all replacement figures and update the figure caption with source information. If applicable, please specify in the figure caption text when a figure is similar but not identical to the original image and is therefore for illustrative purposes only.

The following resources for replacing copyrighted map figures may be helpful:

USGS National Map Viewer (public domain): http://viewer.nationalmap.gov/viewer/

The Gateway to Astronaut Photography of Earth (public domain): http://eol.jsc.nasa.gov/sseop/clickmap/

Maps at the CIA (public domain): https://www.cia.gov/library/publications/the-world-factbook/index.html and https://www.cia.gov/library/publications/cia-maps-publications/index.html

NASA Earth Observatory (public domain): http://earthobservatory.nasa.gov/

Landsat: http://landsat.visibleearth.nasa.gov/

USGS EROS (Earth Resources Observatory and Science (EROS) Center) (public domain): http://eros.usgs.gov/#

Natural Earth (public domain): http://www.naturalearthdata.com/

If the reviewer comments include a recommendation to cite specific previously published works, please review and evaluate these publications to determine whether they are relevant and should be cited. There is no requirement to cite these works unless the editor has indicated otherwise.

Reviewer #1: This manuscript was a pleasure to read. The authors have presented their data well and their arguments are sound. I see no reason that this should not be published quickly with some small additions/modifications that I think will strengthen their paper. I am not best placed to comment on the details of the metagenomic analyses and will leave that to one of the other reviewers. Here my main focus has been on the histological analyses and their interpretation of that and the archaeological evidence.

Please read my comments on the annotated PDF file, there are rather a lot I'm afraid but these are "helpful" suggestions and comments rather than criticisms.

**Corrections based on comments in the attached pdf:**

- In line number 28, “Few” has changed to **“Relatively few”**
- In lines 46-47 the opening sentence has been changed to: **“Bone degradation by microorganisms occurs through enzymatic activities and demineralisation, a process known as bioerosion.”**
- Eriksen et al, 2020 reference is included in citation in the line number 59.
- Lines 65-67: Updated the sentence to **“To our knowledge, this is the first time such detailed histotaphonomic analysis of bioeroded bone samples from archaeological contexts are combined with metagenomic analyses of the bone microbiome.”** as the reviewer pointed out that a similar study has been carried out on experimentally buried bones.
- Line 69: Removed “Wedl” so that the sentence reads “Later scholars…”
- In line number 73 “mineral” has changed to **“matrix”**.
- Line 74-75: Rephrased sentence to: **“So far, however, despite decades of research and new, improved techniques, it has not been possible to confidently identify the microorganisms doing the actual bone bioeroding”**.
- In the lines 130-133 We have expanded the description of the linear regression analysis to clarify variable coding, model structure, and evaluation of model assumptions as: **“Both predictors (Location and Age) were included as categorical variables, with OHI treated as a continuous response variable. Model assumptions were evaluated using standard diagnostic plots, and statistical significance of model terms was assessed using ANOVA.”**
- Line 136: Number of the reference added instead of year, and “in transmitted normal light” removed from the table caption.
- Line 140-143: To expand upon how samples were collected, we added in the Methods section: **“A bone fragment for DNA analysis was collected immediately adjacent to the area used for histological assessment using a Dremel rotary saw fitted with a diamond blade. The excised bone samples (S1 Table) were subsequently powdered, with efforts made, where possible, to maximize the amount of cortical bone while avoiding surface contamination and trabecular bone.”**.
- In the line 208 “OHI” was corrected to **“OHI score”** as suggested.
- Please see the answer to comment #1 by reviewer-1.
- Comment on ‘which Fig 2: This section has been changed, see reply to comment#1 by reviewer-1.
- The original magnification of all images has not been reported, as all images contain a scale. However, a sentence has been added further on the microscopy, lines 121-122: **“…using magnifications between x40 and x400”**.
- Line 234: added **“…reburied outside in their original coffins”**.
- Question regarding evidence of coffin: We’ve added a sentence to the materials description: Lines 92-94: **“All individuals are recovered from soil burials, and in all cases where it could be confirmed, the remains were originally interred in wooden coffins. None were found in intact coffins, and most often only the iron rivets with bits of mineralised wood were preserved.”**
- In the line 298, “environment” was changed to **“burial environment”** to make the statement clear.
- In our dataset, microbial richness correlates with the level of preservation (OHI score), as discussed in the Discussion (lines 435–438). While the chemical/redox characteristics of the grave fill are indeed likely to influence microbial communities, we do not have the necessary screening data to evaluate the effect of redox conditions on microbial diversity in this study.
- Lines 94-97: Added some information on the burial chambers: **“Among the Stavanger cathedral burials, three individuals were originally interred in 17th-century brick-built grave chambers underneath the wood-covered floor of the nave and redeposited in the North graveyard in the 19th century.”**
- Because the detected organisms can only be confidently identified at the genus level, and because post-excavation inoculation of the bones cannot be excluded, it is not possible to reliably infer oxygen availability in these indoor structures based solely on the presence of these organisms.
- Lines 373-374: …low moisture and minimal water movement…, changed to **“…low moisture content…”.**
- Lines 376-380, about effects of metals and humics: We’ve modified the text to suggest these as possible inhibitors of microbial bone degradation, but that the extent of this effect in the cathedral environment is uncertain as: **“Other environmental factors—such as infiltration of humic substances, organic decomposition byproducts, and metal ions from corroding artefacts, have been suggested as inhibitors of microbial bone decay [44,45] —although it is uncertain whether or not this would be substantial enough to explain the low bioerosion in the indoor cathedral remains.”**.
- Lines 382-383: Information added on the indoor metal finds: **“…both due to the rich coffins with metal furnishing, and due to pre-cathedral and construction phase activities”.**
- Comment on hyphae and spores: A sentence and a reference added, lines 460-462: **“Similar shapes of hyphae and spores have been reported by Turner-Walker [21], on the surface of a medieval bone infected by fungi due to poor storage.”**
- We thank the reviewer for raising this point. We have now revised the text in lines 444-447 as **“Members of the genus Lysobacter have been shown through genomic and transcriptomic analyses to encode a diverse repertoire of extracellular lytic enzymes, including proteases with predicted collagenolytic potential, consistent with their role in soil suppressiveness and macromolecule degradation in other environments [50].”**.
- We added in the abstract lines 35-36: **“No fungal species were detected genetically, and histological observations did not suggest any bone degradation by fungi.”**.
- We added in conclusions lines 503-504: **“We found no evidence of fungal degradation, suggesting that the observed bioerosion is mainly caused by bacteria.”**.

**Corrections based on comments of Reviewer#1:**

1) I never like it when I read about "enlarged canaliculi". The diameters of undegraded canaliculi in human bones are really at or beyond the limit of resolution for white light microscopy. For "enlarged canaliculi" I read "more visible canaliculi". The apparent increase in size is either caused by staining which both makes them more visible and alters the refractive index of the surrounding mineralised collagen matrix; or because canaliculi move out of the plane of focus and become visibly broader. In the authors Figure 2E it is the latter. Please see my comment in the caption for Figure 2.

**We agree that it’s an inaccurate term to use and perhaps it should be avoided altogether. We’ve been conservative in assigning this description to the samples, due to the issue that staining may make OCLs and canaliculi ‘more visible’ as the reviewer points out. However, we also see the point of the plane of focus. We’ve modified the text and changed two of the images, and instead of describing it as ‘enlarged’ reporting what Schotsman et al. recently termed ‘onset of degradation’, based on experimental work.**

**We’ve altered and expanded on the original text in line 210-212 in the result section:**

- *The well-preserved areas of bone in many of our samples, including the ones with OHI of 5, exhibited what Schotsman et al. (2024) termed ‘onset of biodegradation’ (Fig 2 A-E) [35].*

**We have also changed images and captions in Fig 2, lines 219-220:**

- *(D) Detail of the same sample, showing possible early-stage bioerosion (red circles), and (E) the same possible onset of biodegradation seen in sample S14393.80.*

2) There are some key points about the burial environments the skeletons were excavated from that appear in the discussion that really should be in the Materials section. I think they should be raised again in the Discussion where they can be related to the authors results. Perhaps a mention of soil water content and probable oxygen availability too if they have that data available to them.

**We do see the reviewer's point. However, we also see now that we forgot to refer to our supplementary document in this part of the materials section. This document provides detailed descriptions of the sites and excavations where the material is from. The skeletons stem from six different cemeteries and ten different excavation campaigns, and there is not enough space to detail this in the main text. Additionally, although Stavanger Cathedral is where we have the most detailed contextual information, and three interesting environments (indoor, outdoor and redeposited), our results summarise the observations across sites. We have, however made some additions and changes to the description of the Stavanger material in the supplementary document.**

**We’ve added the following sentences line 92-97:**

- *“All individuals are recovered from soil burials, and in all cases where it could be confirmed, the remains were originally interred in wooden coffins. None were found in intact coffins, and most often only the iron rivets with bits of mineralised wood were preserved.”*

3) The authors have this at the end of their discussion "Together, the histological and metagenomic evidence suggests that fungi do not play a central role in the bioerosion of bone and that the observed bioerosive patterns are predominantly bacterial in origin." I feel this is such an important conclusion that it should appear in both the Conclusions and the Abstract.

**We’ve added a sentence to the abstract, line 35-36**:

- *“No fungal species were detected genetically, and histological observations did not suggest any bone degradation by fungi.”*

**And to the conclusion, line 502-503:**

- *” We found no evidence of fungal degradation, suggesting that the observed bioerosion is mainly caused by bacteria.”*

4) Also, in the conclusion is this "Due to the nature of the sampled materials, we were unable to confidently assess the effects of post-excavation storage on microbial composition." The authors could add if they intend to tackle this issue in future work (or did I miss that?). Could the authors please add the paper by Provost et al. 2007. Freshly excavated fossil bones are best for amplification of ancient DNA. PNAS 104(3)

**We have no immediate plan to tackle this issue, as our current dataset will not allow us. It would require the analysis of better-controlled and directly comparable skeletal assemblages, excavated at different points in time, or experiments. We are not assessing post-excavation effects on the preservation of human (endogenous) DNA. So, the Pruvost (2007) reference is not relevant here.**

5) The authors make no comment about how their work relates to the "enteric hypothesis" for the origin of bone-degrading bacteria. This may be an intentional omission since they may not want readers to be distracted by such arguments - for which I don't blame them. Best to stick to the data of their own study.

**This is indeed an intentional omission since we can only observe which microorganisms are there, but not say where they come from, whether it’s most likely soil or gut, or a mixture.**

Finally, well done. Nice work.

Reviewer #2: The work addresses an important and topical issue in bioarchaeology and taphonomy – the identification of microorganisms associated with the bioerosion of archaeological bones – and combines histotaphonomy (OHI) with metagenomic aDNA analysis.
More diversity in the microbiome was observed in better-preserved bones
Is it possible to determine whether the microbiome is a ‘remnant of colonisation’ rather than a degrading factor? Also, are heavily eroded bones simply too poor an environment to maintain diversity?

**We added in the lines 449-456 in discussion paragraph:**

- *“Importantly, the observed association between microbial diversity and bone preservation does not allow discrimination between microorganisms acting as active degraders and those representing remnants of past colonisation. Given the cross-sectional and DNA-based nature of the data, the detected microbiome may reflect historical colonization events rather than ongoing bioerosive activity. Alternatively, heavily bioeroded bones may represent a nutritionally and structurally impoverished environment that no longer supports diverse microbial communities. Consequently, the relationships described here should be interpreted as correlative rather than mechanistic.”*

Some subgroups (e.g., redeposited burials, Utstein, Ogna) are very small. In my opinion, it should be noted in Methods/Discussion which comparisons are exploratory in nature, and conclusions based on n ≤ 3 should be formulated more cautiously.
It should be indicated whether the input data for Bray–Curtis was rarefied or normalised using another method.

**We added in lines 108-110 the information as:**

- *“Some site- and context-specific subgroups contained very small sample sizes (n ≤ 3); analyses involving these subsets were therefore considered exploratory and should be interpreted with caution.”*

**We clarified that Bray–Curtis analyses were based on detection-based genus representation rather than read abundance, reflecting a conservative approach appropriate for low-coverage ancient metagenomic data. We updated:**

**Methods part in lines 183-186:**

- *“For community-level analyses, taxonomically authenticated species-level assignments were collapsed to the genus level. For each sample, the number of detected taxa per genus was counted, resulting in a detection-based genus representation rather than a read abundance measure. Genera observed in only a single individual were excluded to reduce noise.”*

**Methods lines 190-195 as:**

- *“Bray–Curtis dissimilarities were calculated from the resulting unrarefied genus-level detection count matrix using the vegdist() function in the vegan package (v2.6.10) in R. Principal coordinate analysis (PCoA) of the Bray–Curtis dissimilarity matrix was performed using the cmdscale() function to visualize patterns of microbiome composition among samples. No rarefaction or read-count-based normalization was applied, as sequencing depth was broadly comparable across samples and read counts are known to be unstable in low-coverage ancient metagenomic datasets”.*

**We further made adaptations to clarify the methodology, results and discussion parts in lines 301 as:**

- *“..based on detection frequency..”*

**Lines 339-342 (Figure caption):**

**“***Fig 6. Microbial composition across different burial contexts.*

*(A) North level 2 (n=9). (B) North level 1 (n=11). (C) North redeposited (n=3), and (D) Nave burials (n=12). For each genus, detections across samples were recorded, and detection frequency was calculated relative to the number of samples within each group.”*

**And lines 431-434:**

- *“Given the instability of read counts across sequencing depths and library preparations in ancient metagenomic datasets, we deliberately focused on detection-based genus-level patterns rather than interpreting read numbers as proxies for microbial abundance.”*

Reviewer #3: Review of the manuscript “ Histological and Metagenomic Analysis of Microbial Communities in Archaeological Human Bones” submitted to Plos One. The manuscript is well-written and structured, and provides interesting insights in characterizing the biodegradation mechanisms affecting archaeological bones. I see a couple of points that show some weakness and I think that they are worth of a deeper discussion, improving the overall strength of the manuscript.
1) Environmental conditions likely play a major role in the microbiome onset; however these are only partially presented and discussed; I suggest to briefly summarize the environmental/climatic data available for the study cases (both current conditions and those encountered during burial, e.g. if climatic/environmental changes occurred in the area during the past centuries.

**While regional paleoclimate reconstructions for Northern Europe indicate broad trends such as the Medieval Warm Period (~950–1250 AD) and the transition toward cooler conditions later in the second millennium, there is no evidence of pronounced, short-term climatic extremes specific to Rogaland between 1000–1800 AD that could be linked directly to individual burial contexts. Moreover, detailed environmental records for the individual excavation sites are not available, preventing analysis of burial-specific climatic or redox influences on microbial communities.**

2) Linked to the previous point, I also suggest crosschecking if any correlation is observed between environmental conditions and OHI, similarly to what is observed in Fig. 3. Statistical analysis should provide evidence on the major factor(s) affecting bone integrity, and if the different environment can explain both the site- and age-specific diagenetic patterns.

**We agree that assessing correlations between environmental conditions and OHI would be highly informative for disentangling the major factors affecting bone integrity. However, detailed and standardized environmental metadata are not available for all excavation sites included in this study, which prevents a robust comparative statistical analysis across contexts.**

**The primary focus of the present manuscript is the relationship between histological preservation (OHI) and microbial community structure. We have therefore limited our analyses to variables that are consistently available across all samples. We added lines 358-359 as:**

*“As a consequence, environmental effects are discussed here qualitatively and at the level of broad burial context (e.g. indoor versus outdoor).”*

3) Page 18, end of second paragraph. I suggest to improve the discussion on the limitations and potential bias occurring when using ancient DNA libraries to detect modern microorganisms.

**We thank the reviewer for this important point. We have expanded the Discussion to explicitly address the limitations and potential bias associated with using ancient DNA libraries to detect microorganisms, including the persistence of DNA from inactive or dead taxa, post-excavation and storage-related colonization, and biases against contemporary microbial communities. We also clarify that DNA-based metagenomic data reflect cumulative microbial presence rather than active degradation processes. The addition in line 469-476:**

- *“A further limitation of this study is inherent to the use of ancient DNA libraries for characterizing bone-associated microbiomes. Such libraries capture DNA from inactive or dead microorganisms, as well as taxa introduced during excavation, handling, or long-term storage, and therefore represent a cumulative record of microbial presence rather than a snapshot of active communities. In addition, aDNA protocols are optimized for fragmented and damaged molecules, which may bias detection toward older or more persistent DNA and underrepresent contemporary microorganisms. These factors complicate the distinction between microbes involved in in situ bone degradation and those reflecting post-depositional or post-excavation processes.”*

Minor comments:
- Page 9, lines 3-5: the sentence is unclear, please rephrase

**We’ve simplified the sentence in lines 229-230:**

- “Possible biofilm was also observed in the microscope. Light and electron images of examples can be seen in S3 Fig.”

- Please mark figure 3 with A, B, C, D

**Figure 3 is marked with A, B, C, D.**

Reviewer #4: The manuscript is clearly structured, presented in an intelligible manner, and written in standard English suitable for an international scientific audience.
The authors confirm that all data underlying the findings will be made fully available upon publication. Sequencing data and associated metadata will be deposited in an appropriate public repository, and accession numbers/DOIs will be provided at that stage. Additional relevant data will be included within the manuscript and its Supporting Information files. Therefore, all data underlying the findings in their manuscript are not fully available. Please make these data available before acceptance.

**We have deposited all raw sequencing data (FASTQ files) in the European Nucleotide Archive (ENA) under the study accession PRJEB104929. The dataset is currently under controlled access and will be made publicly available upon publication of the manuscript, in accordance with journal and ENA data-sharing policies. The accession number has been added to the Data Availability statement in the revised manuscript.**

Once the authors have ensured that all data underlying the findings are made fully available, I strongly recommend the publication of manuscript PONE-D-25-63821 in PLOS ONE, as it presents a robust and innovative methodological framework by combining high-resolution histotaphonomic analyses with metagenomic profiling in a substantial set of medieval and post-medieval human bones. The study establishes quantitative correlations between the Oxford Histological Index (OHI), microbial diversity and environmental variables (burial age, indoor/outdoor context), using appropriate statistical approaches (linear regression, PERMANOVA, alpha and beta diversity analyses), and the results are consistent and well substantiated. The identification of distinct microbial profiles associated with different degrees of bioerosion, with a particular emphasis on the recurrent role of Streptomyces and Streptosporangium, makes a significant contribution to our understanding of bone degradation processes and to the ongoing discussion on the “museum bone microbiome”. The manuscript is clearly structured, well written, engages critically with both classical and recent literature, and offers relevant implications for taphonomy, conservation of osteological collections and ancient DNA studies, fully meeting the scope and methodological standards of rigour expected for PLOS ONE.
