# Supplementary material for: Histological and metagenomic analysis of microbial communities in archaeological human bones
Source: PLoS One. 2026 May 27;21(5):e0340244. doi: 10.1371/journal.pone.0340244 (PMC13215491; doi:10.1371/journal.pone.0340244)
Supplement: Attachment — Submitted filename: Response_to_reviewers_2.docx [file pone.0340244.s016.docx]

Journal Requirements:

1. If the reviewer comments include a recommendation to cite specific previously published works, please review and evaluate these publications to determine whether they are relevant and should be cited. There is no requirement to cite these works unless the editor has indicated otherwise.

**We have carefully reviewed the reviewer comments and confirm that no specific additional references were required. All cited literature has been selected based on its relevance to the study.**

2. Please review your reference list to ensure that it is complete and correct. If you have cited papers that have been retracted, please include the rationale for doing so in the manuscript text, or remove these references and replace them with relevant current references. Any changes to the reference list should be mentioned in the rebuttal letter that accompanies your revised manuscript. If you need to cite a retracted article, indicate the article’s retracted status in the References list and also include a citation and full reference for the retraction notice.

**We have thoroughly reviewed the reference list to ensure that it is complete and accurate. No retracted articles are included. Minor updates and corrections to the reference list have been made where necessary and are reflected in the revised manuscript.**


Reviewers' comments:

6. Review Comments to the Author

Reviewer #1: The authors have done a good job of answering all of the reviewers' comments - either by making changes or by defending their original position. They are to be commended.

One nit-picking comment. The figure text for Figure 2 and Lines 219-220 should read "red ellipses". Yes, I am a pedant!

One last thing. Just after submitting my review of the first version of this paper I came across the attached illustration of Streptomyces sp. online. I think it bears a remarkable similarity to the authors' Figure 2F & G.

**We thank the reviewer for their positive evaluation of our manuscript and for their helpful comments.**

**The suggested correction has been implemented, and the figure text now reads “red ellipses” (Figure 2; Line 219 of the revised manuscript).**

**We also appreciate the reviewer’s observation regarding the similarity between the provided *Streptomyces* illustration and our Figure 2F & G. While this resemblance is interesting, we have refrained from making a direct taxonomic or functional attribution based solely on morphological similarity.**

Reviewer #2: The revised version of the manuscript shows a marked improvement over the previous version. The authors have thoroughly addressed most of the reviewers’ comments, particularly by clarifying methodological aspects and expanding the discussion of the study’s limitations. The changes made have significantly improved the clarity and overall quality of the work.
The study is technically sound, with an adequate sample size and a properly applied approach combining histological and metagenomic analysis. The statistical analyses are appropriate, and the results are presented in a clear and organized manner.
Only minor issues regarding the interpretation of results remain. In particular, given the correlational nature of the data and the use of metagenomic methods, it would be advisable to consistently formulate conclusions in terms of associations rather than the direct functional involvement of specific microbial taxa. Similarly, the interpretation regarding the presence of fungi could be slightly clarified. I am referring to reflecting the discrepancies between microscopic observations and the results of metagenomic analyses.
However, these are editorial issues and can be easily corrected through minor changes in the Discussion and Conclusions section.
In summary, the manuscript is of high quality and represents a valuable contribution to research in this field. I believe that after minor revisions, it will be suitable for publication.

**We thank the reviewer for their positive and constructive evaluation of our manuscript. We appreciate their recognition of the improvements made in this revised version.**

**In response to the reviewer’s comments regarding the interpretation of the results, we have revised the Abstract, Discussion and Conclusions to ensure that our findings are consistently framed in terms of associations rather than direct functional involvement of specific microbial taxa. Statements implying causality have been carefully rephrased throughout the manuscript (e.g. Lines 25, 26, 347, 369, 374-375, 404-405, 432, 437, 438, 440, 441, 444, 445, 453-456, 481, 483, 491, 494, 497).**

**We have also clarified the interpretation of fungal presence by explicitly addressing the discrepancy between histological observations and metagenomic results. The revised text now highlights the potential influence of sampling, preservation, and methodological factors—such as surface cleaning and biases inherent to ancient DNA library preparation—on the detection of fungal DNA (e.g. Lines 35-37, 460, 467-473, 498-499).**

**These revisions have been incorporated into the Discussion and Conclusion sections of the manuscript. We hope that these changes adequately address the reviewer’s concerns.**

Reviewer #3: (No Response)

Reviewer #4: The authors have adequately addressed the comments raised in the previous round of review, and I now consider the manuscript acceptable for publication. It is technically sound, with the data fully supporting the conclusions. Following revision, I have verified that the statistical analyses were performed appropriately and rigorously. The authors have made all underlying data fully available. As a non-native English speaker, I am not best placed to assess this aspect comprehensively; nevertheless, the manuscript is presented intelligibly and in standard English.

In general, I recommend publication.

**We thank the reviewer for their positive evaluation of our manuscript and for recommending its publication. We appreciate their careful assessment and are pleased that the revisions have addressed the previous concerns.**
